# Multi‐Trait Genetic Insights Into Schizophrenia Across Ancestries: Genome‐Wide Association Meta‐Analyses, Machine Learning, and Drug Repurposing Study

**DOI:** 10.1002/brb3.71594

**Published:** 2026-07-09

**Authors:** Ping Yan, Haili Wang, Ye Shu, Rui Xie, Ainiwaner Reyidan, Songnian Fu

**Affiliations:** ^1^ Psychiatric Medical Center The First Affiliated Hospital of Xinjiang Medical University Urumqi China; ^2^ Psychiatry and Mental Health Xinjiang Medical University Urumqi China

## Abstract

**Background:**

Schizophrenia is a psychiatric disorder characterized by a wide range of symptoms and a complex interplay between genetic and environmental factors. Despite advances in genomics, the genetic underpinnings of SC and its relationship with drug use remain poorly understood.

**Methods:**

This study utilized genome‐wide association data from 53,386 SC patients and 77,258 controls of mainly European ancestry, and 22,778 patients and 35,362 controls of mainly East Asian ancestry, alongside GWAS summary data for 23 types of drug use. Genetic correlation analysis was conducted using linkage disequilibrium score regression, followed by meta‐analysis of genome‐wide association studies across multiple traits related to SC. Independent risk loci were identified, and their biological significance was explored using molecular methodologies. Machine learning techniques were employed to develop predictive models for SC diagnosis, and drug repurposing opportunities were investigated.

**Results:**

We found significant genetic correlations between SC and drug use in both ancestries, uncovering 24 previously unidentified loci associated with SC. Through various analytical strategies, we highlighted novel genes linked to SC and developed diagnostic models. Drug repurposing analysis identified ten drug classes with potential therapeutic relevance for SC, including cannabinoid receptor antagonists, which have shown promise in alleviating symptoms.

**Conclusion:**

Our findings reveal a common genetic foundation linking susceptibility to schizophrenia with drug use, providing new insights into the genetic architecture of SC across ancestries. The identification of novel loci and genes associated with SC enhances our understanding of its biological underpinnings and offers potential targets for therapeutic intervention. Additionally, our study opens avenues for drug repurposing as a promising strategy for SC treatment, underscoring the importance of integrating genetic and pharmacological research in psychiatry.

## Graphical abstract

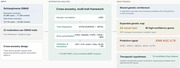

## 1 Introduction

Schizophrenia (SC) presents a challenging landscape in psychiatric disorders, marked by diverse symptomatology and a complex etiology that intertwines significant genetic components with environmental influences (Jablensky 2010). Characterized by a spectrum of positive symptoms, such as delusions and hallucinations; negative symptoms, including loss of motivation and social withdrawal; and cognitive deficits (Owen et al. 2016), SC incurs a profound societal and health impact. It affects approximately 1 in 10,000 adults annually, significantly elevates suicide risks—ranking as the 13th leading cause of suicide globally (Häfner and an der Heiden 1997; Lozano et al. 2012)—and notably diminishes life expectancy by 10–20 years (Chesney et al. 2014).

The pathogenesis of SC is believed to involve a myriad of factors, including genetic, biochemical, environmental, and psychosocial components (Stilo and Murray 2019; Jauhar et al. 2022; Chand et al. 2022). Advances in genomics and bioinformatics have enabled the exploration of genetic variations linked to SC, particularly through genome‐wide association studies (GWAS), which are pivotal for deciphering the biological underpinnings of the disease and fostering the development of novel therapeutic strategies (Sekula et al. 2016; Kiltschewskij et al. 2024; Warren et al. 2024; Bigdeli et al. 2024). Despite previous research indicating common genetic denominators between SC and various drug uses, these studies often lean towards Mendelian randomization (MR) or focus narrowly on the association of specific drug‐related genes with SC (Chauquet et al. 2021; Cheng et al. 2023; Zhuo et al. 2024). The overall genetic correlation—whether it is derived from distinct loci or the genome at large—and the causality and extent of genetic overlap between these phenotypes are yet to be fully determined (Y. Zhang et al. 2021). This underscores the necessity for employing polytrait analysis methods that broaden the phenotypic spectrum under investigation (Chung et al. 2014; C. H. Lee et al. 2021).

**FIGURE 1:**
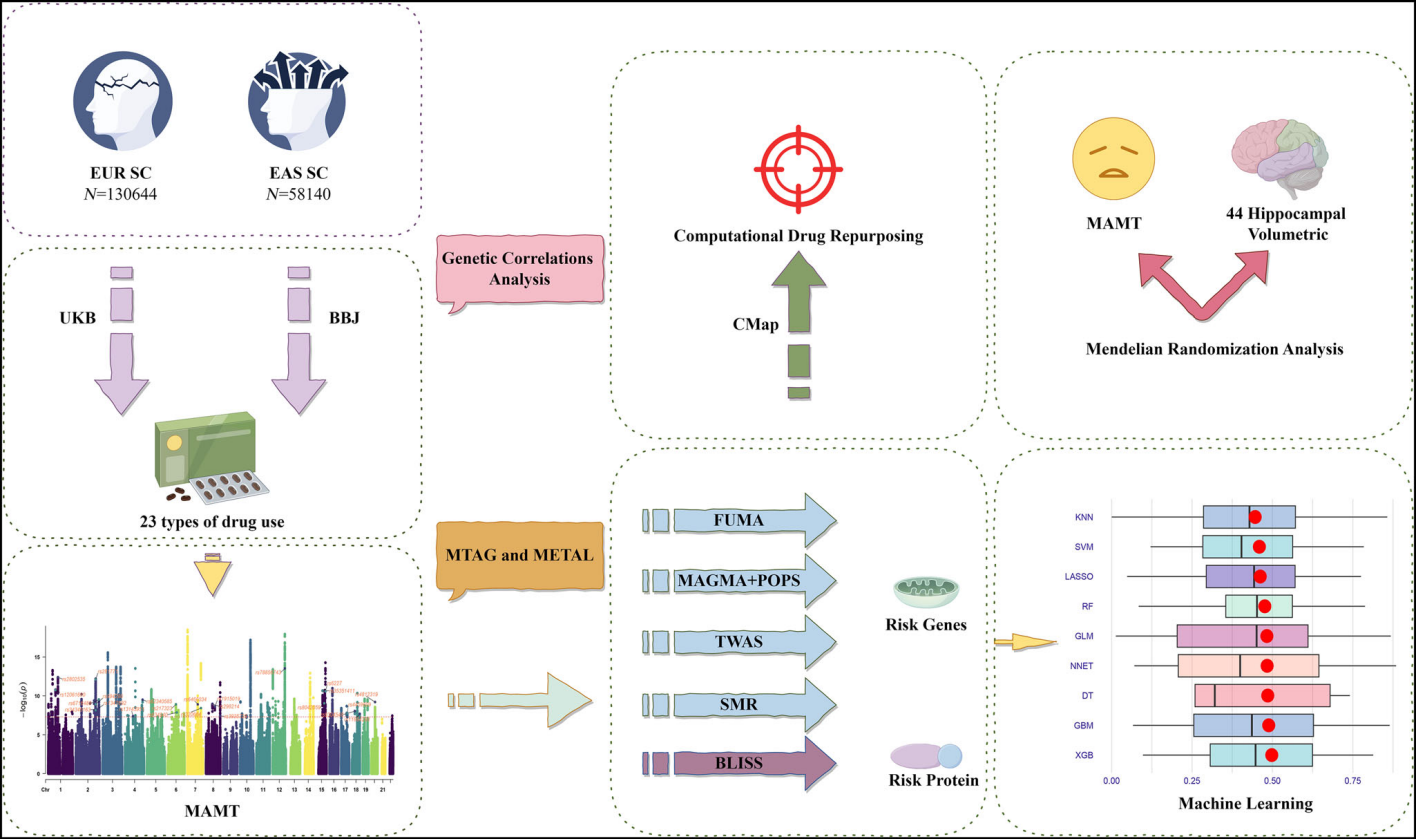
Flowchart. CMap, connectivity map; MAMT, multiple ancestries and traits; MGAMA, multi‐marker analysis of genomic annotation; POPS, polygenic priority score; SC: schizophrenia; SMR, summary data‐based mendelian randomization; TWAS, transcriptome‐wide association.

To exploit existing data resources effectively and enhance the robustness and efficiency of our analyses, we compiled a comprehensive SC GWAS dataset from diverse European and East Asian ancestries. Alongside, we collated GWAS datasets for 23 specific drug uses to explore the shared genetic foundation between SC and drug use. Through multi‐ancestry and multi‐trait meta‐analyses, we sought to unravel the biological mechanisms underpinning SC by conducting in‐depth analyses of risk loci and employing molecular‐based association studies (Figure 1).

## 2 Methods

### 2.1 Genetic Correlation Analysis

Our analysis incorporated data from the most comprehensive GWAS on SC to date. This included data from 53,386 SC patients and 77,258 controls of mainly European ancestry (MA EUR), along with 22,778 patients and 35,362 controls of mainly East Asian ancestry (MA EAS) (Trubetskoy et al. 2022; Lam et al. 2019). We also utilized GWAS summary data for the use of 23 different drugs from the Pan‐UK Biobank and BioBank Japan PheWeb (Tables S1–S2) (Rusk 2018; Sakaue et al. 2021).

To assess the genetic correlations between SC and the 23 drug uses across European and East Asian populations, we employed the linkage disequilibrium score regression (LDSC) methodology (Bulik‐Sullivan et al. 2015). This analysis was underpinned by the linkage disequilibrium (LD) scores derived from the 1000 Genomes Project for both European and East Asian ancestries (Auton et al. 2015). Our LDSC analysis did not enforce an intercept constraint, acknowledging that while sample overlap might influence the intercept value, it does not affect the slope estimate, and hence, the genetic relationship estimation remains intact. This approach enables the identification of potential residual confounders and assesses the presence of sample overlap between GWAS datasets. Genetic correlations were deemed significant at a false discovery rate (FDR) of 0.05, based on the p‐value for genetic correlation.

### 2.2 Meta‐Analysis of GWAS

To investigate genome‐wide associations across multiple traits (MT) related to SC and its genetically correlated traits across different ancestries, we utilized the multiple trait analysis of GWAS (MTAG) software. This tool is designed to enhance the detection of novel genetic associations by leveraging the correlation among traits, thus improving statistical power. Specifically, MTAG performs a generalized inverse variance weighted (IVW) meta‐analysis on the summary statistics from single‐trait GWAS, producing trait‐specific effects for a shared set of SNPs for European (MT EUR) and East Asian ancestries (MT EAS) separately (Turley et al. 2018; Guo et al. 2022).

For the analysis encompassing multiple ancestries and traits (MAMT), we adopted the IVW fixed‐effects model using METAL software. This approach integrates results from MT analyses across ancestries, providing a comprehensive view of genetic associations (Willer et al. 2010; Han and Eskin 2011). We also conducted heterogeneity analyses with METAL to evaluate the consistency of effect sizes across different samples.

Consistent with protocols for autoimmune diseases, all meta‐analyses excluded data from the Major Histocompatibility Complex (MHC) region on chromosome 6 (25–35 MB) due to its large effect sizes, which could bias the analysis by violating MTAG's modeling assumptions (Khunsriraksakul et al. 2023).

### 2.3 Cross‐Ancestry Genetic Correlations

Our analysis extended to evaluating the genetic correlations across ancestries, specifically between SC of MA EUR and MA EAS ancestries, as well as between the multi‐trait analyses results for these ancestries (MT EUR and MT EAS), using the POPCORN tool. POPCORN is designed to work with summary‐level data from GWAS, leveraging SNP associations across the entire genomic spectrum. This method addresses computational efficiency and privacy concerns by obviating the need for individual‐level genotype data. It is adept at handling analyses involving large cohorts, up to hundreds of thousands of individuals, and millions of SNPs (Brown et al. 2016). For our purposes, we utilized cross‐population scores pre‐calculated by POPCORN, which are tailored to the European and East Asian populations based on the 1000 Genomes Project data (Brown et al. 2016).

### 2.4 Identification of Independent Risk Loci

To identify independent genetic risk loci, we used the Functional Mapping and Annotation of Genetic Associations (FUMA) platform with a genome‐wide significance threshold of *p* < 5 × 10^−8 31^. FUMA was used to define genomic risk loci and annotate associated variants based on LD information from the 1000 Genomes Project Phase 3 reference panel for populations of mixed ancestry. Lead single nucleotide variants (SNVs) were defined as independent genome‐wide significant variants with *p* < 5 × 10^−8^. SNVs with p < 0.05 within the corresponding genomic regions were retained as candidate variants for functional annotation but were not considered genome‐wide significant. Independent significant SNVs were defined using an LD threshold of *r* ^2^ < 0.2, whereas lead SNVs were further defined by *r* ^2^ < 0.1 within a 1‐Mb window. Genomic risk loci were constructed by merging regions in which the distance between neighboring lead SNVs was less than 500 kb (Gong et al. 2023).

Annotation of these SNVs was performed using ANNOVAR, integrating combined annotation‐dependent depletion (CADD) scores and RegulomeDB evaluations to assess the functional impact of each variant (K. Wang et al. 2010; Rentzsch et al. 2019; Dong et al. 2023).

To ensure the replicability of identified independent risk loci across ancestries, we employed a statistical fine mapping strategy that extends Bayesian fine mapping (Lam et al. 2019). This approach accounts for heterogeneity across ancestral populations, assigning lower prior probabilities of causality to variants with inconsistent effect estimates across groups (Gormley et al. 2016). For each locus, a 99% confidence set was established by ordering SNPs (within an *r* ^2^ > 0.2 of the target variant) by their posterior probabilities, continuing until the cumulative probability reached or surpassed 0.99 (Meng et al., 2024).

### 2.5 Gene Annotation and Association Analysis

Gene annotation of MAMT summary statistics was performed using FUMA, based on both positional mapping and expression quantitative trait locus (eQTL) mapping. The 1000 Genomes Project Phase 3 reference panel was used as the LD reference for FUMA annotation. For eQTL mapping, we used all GTEx v8 brain tissue datasets implemented in FUMA to improve the biological relevance of gene annotation in neurological contexts (Watanabe et al. 2017; Meng et al. 2024).

We further performed gene‐based association analyses using multi‐marker analysis of genomic annotation (MAGMA) and polygenic priority score (POPS) methods (de Leeuw et al. 2015; Weeks et al. 2023). For MAGMA, statistical significance was determined using a Bonferroni‐corrected threshold based on the number of genes tested, defined as *p* < 2.70 × 10^−6^ (0.05/18,522). POPS was subsequently applied for gene prioritization by integrating genome‐wide GWAS summary statistics with public functional genomics resources, including bulk and single‐cell expression profiles, curated biological pathways, and protein‐protein interaction predictions (Weeks et al. 2023). Gene‐level statistics derived from MAGMA were used as input for POPS analysis, and genes with a POPS score >1 were retained as prioritized genes rather than being interpreted as statistically significant genes.

### 2.6 Transcriptome‐Wide Association Analysis

We conducted transcriptome‐wide association analysis (TWAS) using FUSION, applying SNP‐expression weights derived from multiple external transcriptomic reference panels. These included prediction weights for brain tissues, adrenal and pituitary glands, thyroid, mammary gland, and whole blood, primarily from GTEx v8 and supplemented by weights from the CommonMind Consortium (Dall'Aglio et al. 2021; Gusev et al. 2016). To account for the ancestry composition of the GWAS summary statistics and reduce potential bias caused by LD mismatch, we used a multi‐ancestry LD reference panel in FUSION. This LD reference panel consisted of 984 individuals of EUR and EAS ancestry. A transcriptome‐wide significance threshold was defined using Bonferroni correction as *p* < 1.93 × 10^−6^ (0.05/25,931), where 25,931 represents the number of genes tested in the TWAS.

### 2.7 Gene‐Phenotype Correlation Analysis

We investigated the correlations between genes and SC through summary data‐based MR analysis (SMR), leveraging MAMT and eQTL data from diverse sources, including brain tissue (BrainMeta), blood (eQTLGen consortium), and nine brain cell types (Qi et al. 2022; Võsa et al. 2021; Bryois et al. 2022; Jerber et al. 2021). SMR, integrating GWAS and QTL study data, was employed to identify pleiotropic associations between gene expression and SC (Zhu et al. 2016). A multi‐ancestry LD reference panel was utilized for LD estimations. Significance in gene‐trait associations was determined by a Bonferroni‐adjusted SMR *p*‐value < 0.05 and HEIDI test results (*p* > 0.01).

### 2.8 Identification of Reliable Genes

To assemble a set of reliable causal genes, we integrated insights from various gene mapping strategies. Specifically, we combined data from FUMA annotation, POPS, TWAS, and SMR analyses. Genes identified across these three methodologies were prioritized as key targets.

For these target genes, we conducted phenotypic enrichment analysis. Specifically, this analysis was based on the mammalian phenotype (MP) ontology from mouse genome informatics (MGI) (Blake et al. 2021). By examining the differences in the proportion of genes associated with certain phenotypes between pleiotropic and non‐pleiotropic gene sets, we assessed the phenotypic specificity of these genes. The Fisher exact test was utilized to discern significant disparities in phenotype associations within the non‐pleiotropic gene set.

### 2.9 Transcriptome Validation Through Machine Learning

To investigate the expression profiles of target genes in SC patients, we utilized five mRNA expression datasets from the gene expression omnibus (GEO), comprising data from 196 SC patients and 173 healthy controls (Table S3) (Clough and Barrett 2016; Bousman et al. 2010; van Beveren et al. 2012; de Jong et al. 2012; van Eijk et al. 2015; Gatta et al. 2021). To mitigate batch effects across these datasets, we employed the “combat” method within the “sva” package, creating normalized composite expression matrices. The efficacy of batch effect removal was confirmed through box plots and principal component analysis (PCA) (Johnson et al. 2007).

Further, we applied a suite of nine machine learning algorithms—including Support Vector Machine, Random Forest, K‐Nearest Neighbor, Gradient Boosting Machine, LASSO, XGBoost, Neural Networks, Generalized Linear Model, and Decision Tree—to prioritize target genes. For each algorithm, we computed the Area Under the Curve (AUC) and generated receiver operating characteristic (ROC) curves to evaluate the performance of gene prioritization.

### 2.10 Computational Drug Repurposing

To identify candidate small‐molecule compounds with the potential to reverse the SC‐associated genetically regulated expression profile, we performed computational drug repurposing (CDR) using the Connectivity Map (CMap) algorithm. TWAS *Z*‐scores were used to construct the query gene expression signature. Specifically, genes with positive TWAS *Z*‐scores were defined as putatively upregulated SC‐associated genes, whereas genes with negative TWAS *Z*‐scores were defined as putatively downregulated SC‐associated genes. These signed gene sets were then queried against the CMap database to identify perturbagens whose induced expression profiles were negatively connected with the SC‐associated signature.

The CMap database contains gene expression signatures generated from human cell lines exposed to pharmacological and genetic perturbations, thereby enabling systematic comparison between disease‐associated gene expression signatures and compound‐induced perturbation profiles. Our analysis was restricted to the CMap Touchstone dataset, which includes reference signatures from nine human cell lines treated with approximately 3000 well‐characterized small‐molecule compounds (Khunsriraksakul et al. 2023; Z. Wang et al. 2018).

CMap uses the *τ*‐score to quantify the concordance between the query signature and reference perturbation signatures. A negative *τ*‐score indicates that the compound‐induced expression profile is inversely correlated with the SC‐associated query signature, suggesting a potential ability to counteract or normalize trait‐associated gene expression changes. Therefore, compounds with more negative *τ*‐scores were prioritized as candidate drugs for potential repurposing in SC treatment (Khunsriraksakul et al. 2023; Z. Wang et al. 2018).

### 2.11 Protein‐Based Association Analysis

To delineate the proteomic profile associated with SC, we utilized the Biomarker expression Level Imputation using Summary‐level Statistics (BLISS) method. Unlike traditional proteome‐wide association studies (PWAS) that depend on individual‐level reference proteomes, BLISS leverages summary‐level pQTL data, enabling the use of extensive public domain datasets. We trained proteomic models for European populations using pQTL information from the UK Biobank, deCODE, and ARIC studies, systematically validating these models across multiple ancestries to ensure their efficacy (C. Wu, Zhang, Yang, et al. 2023). Proteins identified with Bonferroni‐adjusted *p* < 0.05 were considered significant risk factors.

### 2.12 MR Analysis

A bidirectional two‐sample MR analysis was conducted to investigate the potential causal link between SC and 44 hippocampal volumetric traits. Utilizing the TwoSampleMR, we adhered to a genome‐wide significance threshold of *p* < 5 × 10^−8^, with LD criteria set at *r* ^2^ < 0.01 and distance greater than 5000 kb (Lv et al. 2023). IVW estimates were chosen as the primary analytic outcome due to their robust statistical power. The MR Egger intercept was employed to assess the presence of pleiotropy among instrumental variables (Bowden et al. 2017). Our analysis included a genome‐wide association meta‐analysis for hippocampal and subregional volumes across European and East Asian ancestries, featuring 7009 individuals of East Asian descent (Liu et al. 2023; Xu et al. 2020). We identified 339 significant associations (*p* < 1.13 × 10^−9^) for the 44 hippocampal traits (Table S4) (Xu et al. 2020).

## 3 Results

### 3.1 Meta‐Analysis Findings

LDSC analysis identified significant genetic correlations between SC and several drug use traits in both MA‐EUR and MA‐EAS populations after FDR correction. In the MA‐EUR population, seven drug use traits showed significant genetic correlations with SC, including drugs used in diabetes, diuretics, beta blocking agents, agents acting on the renin‐angiotensin system, HMG CoA reductase inhibitors, anilides, and antidepressants. Among these, drugs used in diabetes, diuretics, beta blocking agents, agents acting on the renin‐angiotensin system, and HMG CoA reductase inhibitors showed negative genetic correlations with SC, whereas anilides and antidepressants showed positive genetic correlations.

**FIGURE 2:**
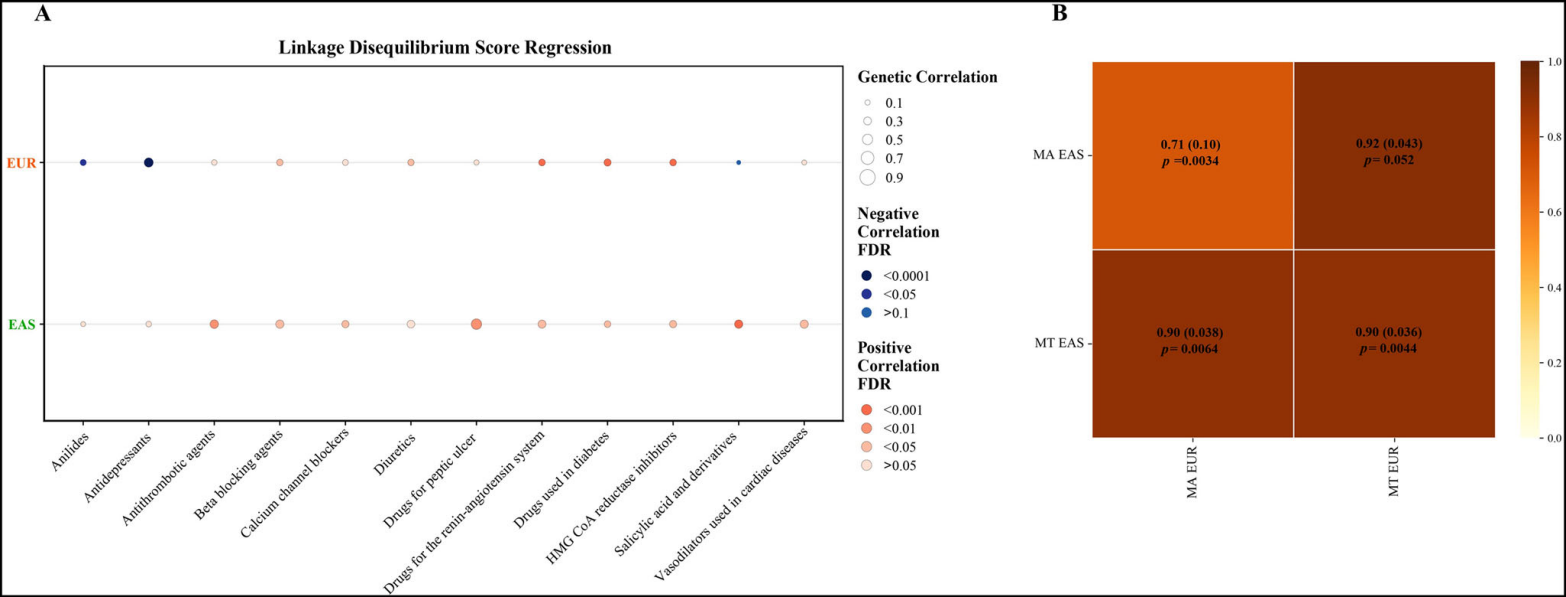
Genetic correlation analysis of schizophrenia with drug use across ancestries. (A) Genetic correlations between schizophrenia and 23 types of drug use in European and East Asian ancestries analyzed using LDSC (linkage disequilibrium score regression) with data from the UK Biobank (UKB) and BioBank Japan (BBJ). Larger circles denote stronger genetic correlations, whereas deeper colors indicate higher significance levels. (B) Cross‐ancestry genetic correlations between schizophrenia in European ancestry (MA EUR) and East Asian ancestry (MA EAS), as well as between multi‐trait analyses results for MT EUR and MT EAS, assessed using the POPCORN tool.

In the MA‐EAS population, nine drug use traits showed significant genetic correlations with SC, including drugs for peptic ulcer and gastroesophageal reflux disease, drugs used in diabetes, antithrombotic agents, vasodilators used in cardiac diseases, beta‐blocking agents, calcium channel blockers, agents acting on the renin‐angiotensin system, HMG CoA reductase inhibitors, and salicylic acid and derivatives. All nine significant correlations in MA‐EAS were negative. Four drug use traits, including drugs used in diabetes, beta blocking agents, agents acting on the renin‐angiotensin system, and HMG CoA reductase inhibitors, were significantly and negatively correlated with SC in both MA‐EUR and MA‐EAS populations (Table 1; Figure 2A).

**TABLE 1:** Genetic correlation analysis of schizophrenia with 23 types of drug use in European and East Asian Ancestries Using LDSC.

Phenotype	EUR				EAS			
	Genetic correlation	Z score	*p* value	FDR	Genetic correlation	Z score	*p* value	FDR
Drugs for peptic ulcer and gastroesophageal reflux disease	−0.0362 (0.0276)	−1.3135	1.89E‐01	3.62E‐01	−0.3325 (0.0987)	−3.3671	8.00E‐04	5.87E‐03
Drugs used in diabetes	−0.1119 (0.027)	−4.1451	3.40E‐05	3.91E‐04	−0.0862 (0.0354)	−2.4349	1.49E‐02	4.42E‐02
Antithrombotic agents	−0.0446 (0.0292)	−1.5277	1.27E‐01	2.65E‐01	−0.1875 (0.0534)	−3.5141	4.00E‐04	4.40E‐03
Vasodilators used in cardiac diseases	−0.0299 (0.0444)	−0.6727	5.01E‐01	6.40E‐01	−0.1695 (0.0589)	−2.8778	4.00E‐03	1.76E‐02
Antihypertensives	−0.0501 (0.0454)	−1.1032	2.70E‐01	4.78E‐01	−0.1326 (0.0681)	−1.9478	5.14E‐02	1.03E‐01
Diuretics	−0.0768 (0.0261)	−2.9425	3.30E‐03	1.27E‐02	−0.1379 (0.0709)	−1.9453	5.17E‐02	1.03E‐01
Beta blocking agents	−0.0806 (0.0272)	−2.9591	3.10E‐03	1.27E‐02	−0.1712 (0.0675)	−2.535	1.12E‐02	4.11E‐02
Calcium channel blockers	−0.0553 (0.0243)	−2.2778	2.27E‐02	6.53E‐02	−0.1147 (0.0478)	−2.4003	1.64E‐02	4.42E‐02
Agents acting on the renin‐angiotensin system	−0.0881 (0.0229)	−3.8497	1.00E‐04	5.75E‐04	−0.1597 (0.0521)	−3.0661	2.20E‐03	1.21E‐02
HMG CoA reductase inhibitors	−0.0902 (0.0236)	−3.8156	1.00E‐04	5.75E‐04	−0.1152 (0.0487)	−2.3642	1.81E‐02	4.42E‐02
Thyroid preparations	0.011 (0.0238)	0.4622	6.44E‐01	7.40E‐01	−0.0015 (0.0732)	−0.0202	9.84E‐01	9.84E‐01
Immunosuppressants	−0.0365 (0.0647)	−0.5641	5.73E‐01	6.93E‐01	0.0183 (0.07)	0.2617	7.94E‐01	8.73E‐01
Anti‐inflammatory and antirheumatic products, non‐steroids	−0.0611 (0.0285)	−2.1436	3.21E‐02	8.20E‐02	−0.2231 (0.1208)	−1.8463	6.49E‐02	1.19E‐01
Drugs affecting bone structure and mineralization	0.0358 (0.0408)	0.8781	3.80E‐01	5.46E‐01	−0.1297 (0.0832)	−1.5593	1.19E‐01	2.01E‐01
Opioids	−0.0073 (0.0268)	−0.2739	7.84E‐01	8.01E‐01	NA	NA	NA	NA
Salicylic acid and derivatives	0.0225 (0.0323)	0.6982	4.85E‐01	6.40E‐01	−0.1734 (0.0419)	−4.1366	3.52E‐05	7.74E‐04
Anilides	0.083 (0.0295)	2.8171	4.80E‐03	1.58E‐02	−0.0276 (0.2535)	−0.109	9.13E‐01	9.57E‐01
Antimigraine preparations	0.0144 (0.0361)	0.3991	6.90E‐01	7.55E‐01	0.2008 (0.1594)	1.2598	2.08E‐01	3.26E‐01
Antidepressants	0.2542 (0.0333)	7.6242	2.46E‐14	5.66E‐13	−0.0493 (0.1276)	−0.3866	6.99E‐01	8.09E‐01
Adrenergics, inhalants	0.0265 (0.0272)	0.9759	3.29E‐01	5.17E‐01	−0.0634 (0.0738)	−0.8592	3.90E‐01	5.37E‐01
Glucocorticoids	0.0326 (0.034)	0.9592	3.37E‐01	5.17E‐01	−0.08 (0.079)	−1.0118	3.12E‐01	4.57E‐01
Antihistamines for systemic use	0.0096 (0.0382)	0.2519	8.01E‐01	8.01E‐01	0.0367 (0.0768)	0.478	6.33E‐01	7.73E‐01
Antiglaucoma preparations and miotics	−0.0701 (0.0353)	−1.9858	4.71E‐02	1.08E‐01	−0.0657 (0.1242)	−0.5294	5.97E‐01	7.72E‐01

Abbreviations: BBJ, BioBank Japan; LDSC, linkage disequilibrium score regression; UKB, UK Biobank.

**FIGURE 3:**
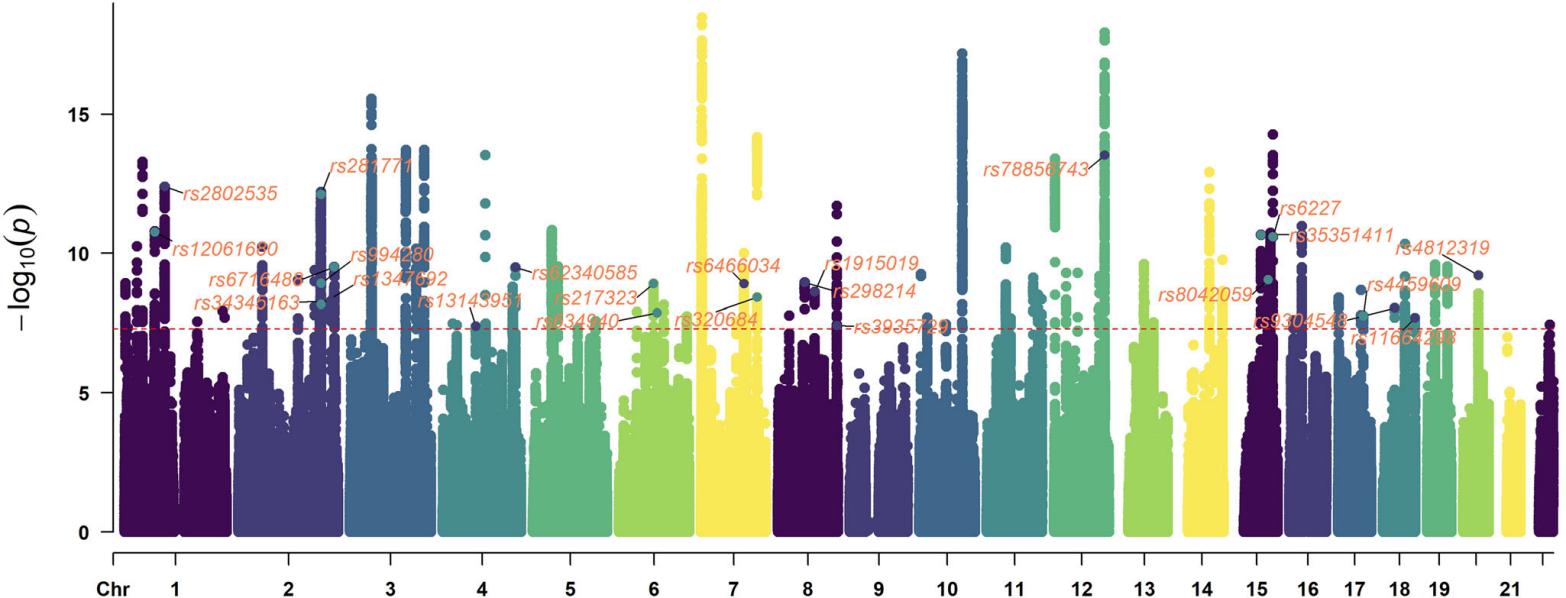
Manhattan plot for MAMT. The plot highlights newly identified single nucleotide variants (SNVs) located more than ±250 kb from previously reported variants, with a significance threshold set at 5 × 10^−8^.

MTAG further delineated these relationships, producing multi‐trait results for MA EUR and MA EAS, consolidated into a combined ancestry meta‐analysis (MAMT). This comprehensive analysis yielded 7,316,997 SNP results without signs of residual population stratification (*λ* = 1.15, LDSC Intercept = 0.69; Figure 3). The POPCORN tool indicated that cross‐ancestry genetic correlations between MT EUR and MT EAS (0.90) exceeded those initially identified between MA EUR and MA EAS (0.71), underscoring a substantial shared genetic foundation among these traits (Figure 2B).

We identified 130 independent genome‐wide significant SNPs across 84 loci, with 24 novel associations discovered beyond ±250 kb from known variants (Figure 3). A Bayesian fine mapping approach validated these SNPs, narrowing down to a 99% confidence set of 126 SNVs without multiple independent signals (Table S6). Notably, METAL's heterogeneity test confirmed the consistency of these findings (Bonferroni threshold 0.05/130).

Annotation with ANNOVAR highlighted 12 SNVs with CADD scores exceeding 12.37. Among the new SNVs, rs6227 in the 15q26.1 region had the highest CADD score (10.19), associating it with trauma exposure measures and the FURIN gene, which was further explored in our subsequent study (Table S5) (Coleman et al. 2020).

### 3.2 Genetic Screening

To elucidate the biological foundations of our GWAS results, we conducted comprehensive computer analyses for functional annotation and gene prioritization. FUMA annotation identified 470 genes as potentially involved in SC, providing a broad overview of genetic underpinnings (Table S7). Separately, MAGMA analysis pinpointed 207 genes, offering another layer of insight into the genetic architecture of SC. Further refinement with the POPS highlighted 22 genes with a POPS score greater than 1, underscoring their potential causative roles in SC (Table S8).

**FIGURE 4:**
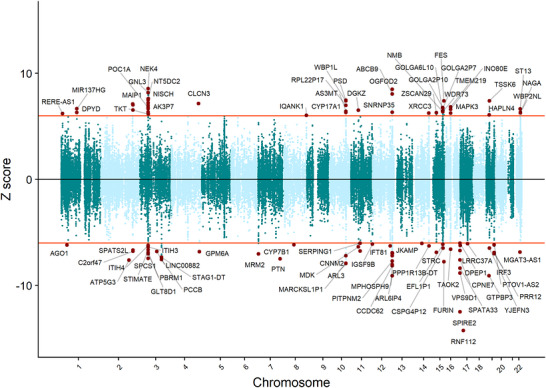
Manhattan plot of *Z*‐scores for genes associated with SC in TWAS. Significant gene associations are marked with red dots, and the names of genes with (*Z* > |7|) are prominently displayed on both sides of the plot. For genes at the top, increased expression is associated with elevated SC risk, whereas genes at the bottom show a negative correlation with expression.

TWAS highlighted 320 significant gene‐SC correlations (*p* < 1.93 × 10^−6^; Table S9). Notably, NEK4 emerged as the most positively correlated gene (*Z* = 8.55, *p* = 1.24 × 10^−17^), exhibiting significant correlations across multiple tissues, including brain regions and peripheral tissues like the spinal cord and thyroid. Conversely, RNF112 was the most negatively associated gene with SC (*Z* = −14.25, *p* = 1.20 × 10^−22^; Figure 4). PCCB showed significant associations in 17 tissues, aligning with prior research (Trubetskoy et al. 2022; W. Zhang et al. 2023).

SMR analysis identified 72 SC‐related genes (Table S10), with OGFOD2 and ACE being the most significantly associated (*β* = 0.81, adj. *p* = 2.02 × 10^−3^ and *β* = −0.48, adj. *p* = 4.82 × 10^−2^). Moreover, we discovered that ARL17B exhibited a significant negative correlation with SC across seven types of brain cells (astrocytes, endothelial cells, excitatory neurons, inhibitory neurons, microglia, oligodendrocytes, and oligodendrocyte progenitor cells), corroborating earlier findings (Y. Wu, Zhang, Wang, et al. 2023).

**FIGURE 5:**
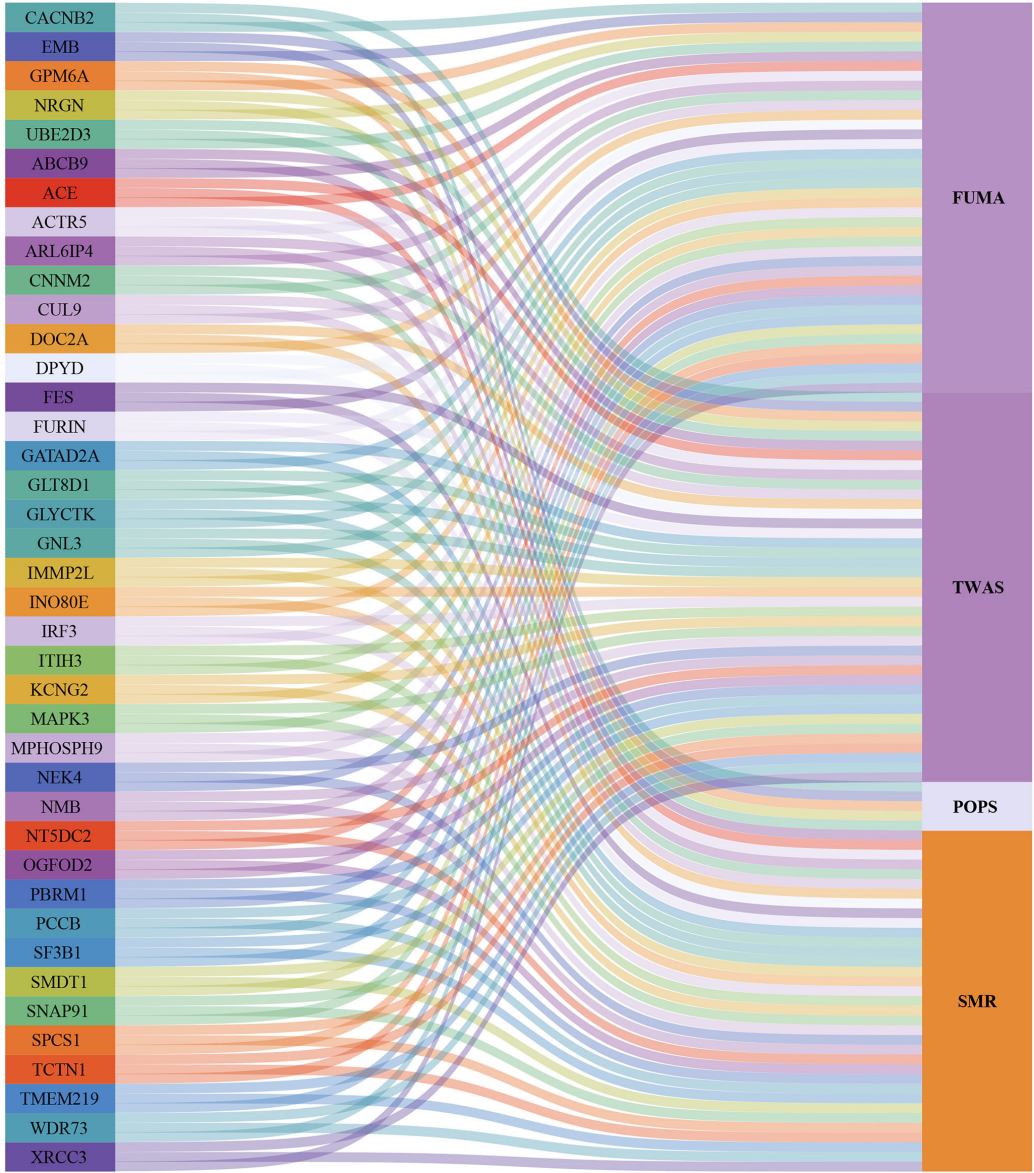
Forty SC risk genes identified using four distinct methodologies, each gene recognized by three or more approaches. The left side represents the risk genes, while the right‐side details the various methods employed.

**FIGURE 6:**
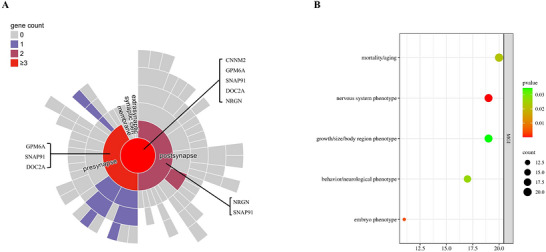
Synaptic gene and phenotype analysis of credible SC genes. (A) Sunburst diagram depicting synaptic locations starting from the synapse (center), positions pre‐ and postsynaptic in the first ring, and subsequent subcategories (i.e., items as subsets of their adjacent inner ring). Locations enriched with credible SC genes and the genes within these locations are marked. The number of genes per term is represented by the color scheme in the legend. (B) Parallel phenotype analysis of credible SC genes.

Integrating results across the four aforementioned methods, we identified 40 high‐confidence genes present in three or more analyses (Figure 5), including previously emphasized genes like MAPK3 and ACE, alongside 26 genes not highlighted in recent large‐scale SC studies (Trubetskoy et al. 2022). Among these, seven genes have synaptic annotations in the SynGO database, indicating their involvement in synaptic functions (Figure 6A) (Koopmans et al. 2019).

Phenotypic analysis of credible SC genes revealed five significant associations with SC (Figure 6B; Table S11). These associations span neurological phenotypes, reflecting the neurobiological basis of SC; growth/size/body region phenotypes, hinting at biological variances in SC patient development; embryonic phenotypes, suggesting the influence of early developmental stages on SC risk; and behavioral/neurological phenotypes, mirroring clinical neurobehavioral symptoms. Additionally, mortality/aging correlations underscore SC's impact on overall health and longevity, highlighting the disease's potential to affect life quality (Jones et al. 2011; Anttila et al. 2018; Brouwer et al. 2022; Kelley and Pașca 2022).

### 3.3 Machine Learning‐Assisted Gene Prioritization

**FIGURE 7:**
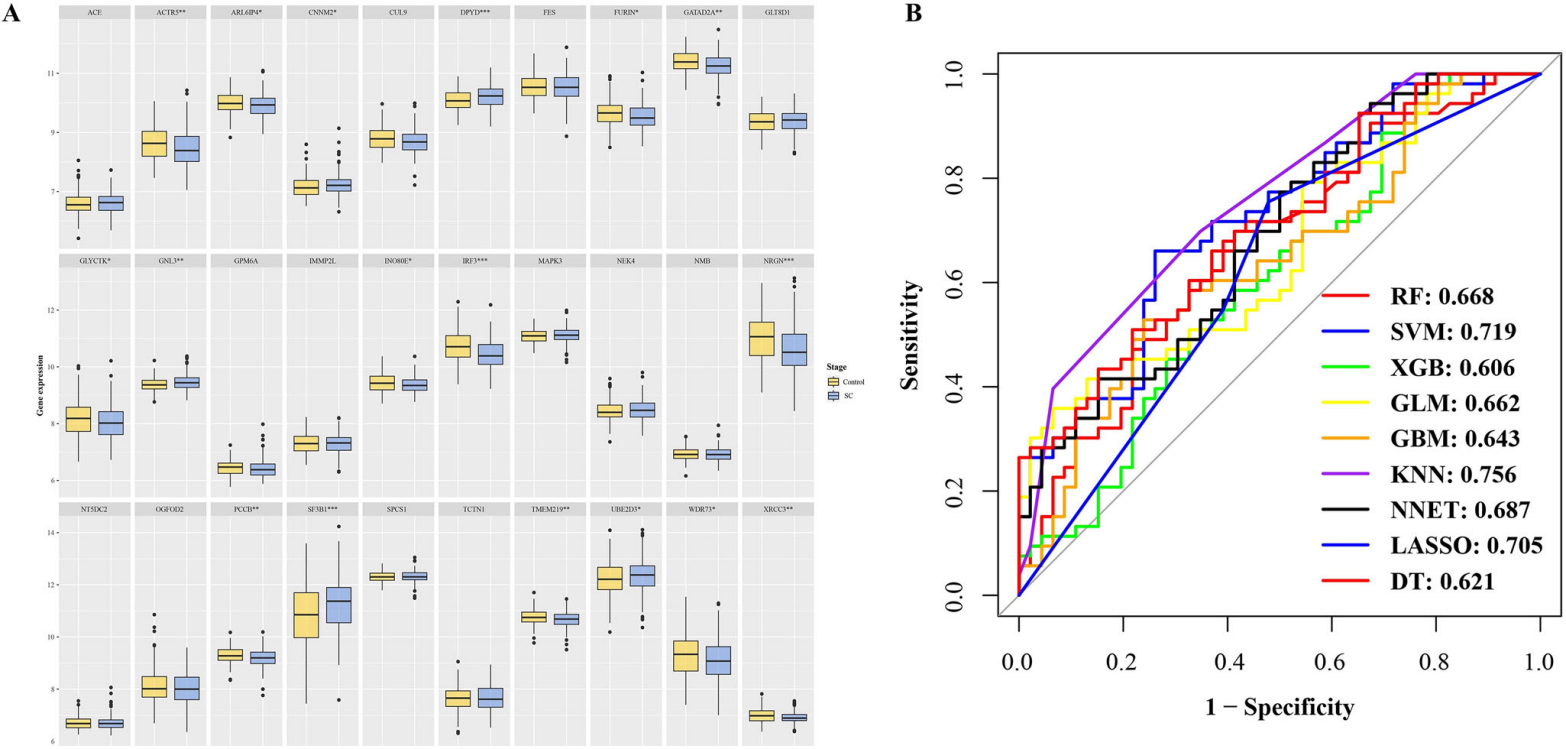
Expression and diagnostic modeling of credible SC genes. (A) Boxplot showing expression differences in transcriptomes for 31 of the 40 credible SC genes (ABCB9 gene excluded for visual clarity). (B) ROC curves for diagnostic models constructed with the 31 credible genes using nine machine learning algorithms. DT, decision tree; GBM, gradient boosting machine; GLM, generalized linear model; KNN, K‐nearest neighbor; NNET, neural networks; RF, random forest; SVM, support vector machine; XGB, XGBoost.

Integrating data from five transcriptomic studies resulted in an analysis set of 10,498 genes, including 31 of the 40 genes deemed plausible for SC involvement. Using the rank‐sum test, 17 of these genes (54.8%) exhibited significant differential expression between SC patients and controls (*p* < 0.05; Figure 7A and Table S12), with IRF3 emerging as the most significant (*p* = 1.5 × 10^−7^).

Subsequently, we applied nine machine learning algorithms to develop an SC diagnostic model (Figure 7B). The K‐Nearest Neighbor (KNN) algorithm achieved the highest discriminative performance (AUC = 0.76), highlighting SF3B1, WDR73, UBE2D3, NRGN, and ACTR5 as key diagnostic genes. Remarkably, IRF3 was consistently scored highest across multiple algorithms (Figure S1), underscoring its potential as a pivotal gene in SC diagnosis.

### 3.4 CDR Analysis

Utilizing TWAS Z‐values for 40 genes implicated in SC, we conducted CDR using the CMap approach. This analysis identified ten drug classes with potential clinical relevance for SC (Table S13). Notable among these were cannabinoid receptor agonists/antagonists, phenylalanyl tRNA synthetase inhibitors, bacterial cell wall synthesis inhibitors, and nucleoside reverse transcriptase inhibitors. Interestingly, certain drugs within these classes, such as cannabinoid receptor antagonists, have previously been reported to mitigate symptoms in SC, including improving sensorimotor gating deficits in phenylcyclohexylpiperidine (PCP)‐induced SC models (Ballmaier et al. 2007).

### 3.5 Protein Association Analysis

Integrating plasma protein data from the deCODE and UKB databases with MAMT via the BLISS approach identified 20 and 16 proteins of interest, respectively (Table S14). Four proteins—ITIH3, ADAM22, PTN, and DPEP1—demonstrated significant associations with SC across both datasets. Specifically, ITIH3 and ADAM22 exhibited negative correlations, whereas PTN and DPEP1 were positively correlated with SC. Noteworthy is that ITIH3, also highlighted among the 40 high‐confidence genes from our study, consistently showed negative correlations in both TWAS and SMR analyses, underscoring its potential relevance to SC pathology.

### 3.6 Mendelian Randomization

Our MR analysis investigated the causal links between MAMT and hippocampal volumes across ancestries (Table S15). IVW analysis indicated a significant bidirectional relationship between right presubiculum volume (RPV) and SC (SC→RPV: *β* = −0.064, *p* = 0.039; RPV→SC: *β* = −0.19, *p* = 0.040), with similar findings for the left and right hippocampal CA3 subregions (Right→SC:*β* = −0.13, *p* = 0.010; Left→SC:*β* = −0.19, *p* = 0.017), without evidence of pleiotropy. These results suggest a potential reflection of SC pathology in specific brain structure volume reductions.

## 4 Discussion

In this study, we integrated SC GWAS data from European and East Asian ancestries with GWAS summary statistics for 23 drug use traits, followed by multi‐trait meta‐analysis, gene prioritization, transcriptomic validation, proteomic association analysis, drug repurposing, and MR. The main findings can be summarized in three aspects. First, SC showed reproducible genetic correlations with several drug use traits across ancestries, suggesting that part of the genetic architecture of SC overlaps with medication‐related phenotypes. Second, cross‐ancestry and multi‐trait meta‐analysis improved locus discovery and highlighted a set of biologically plausible genes supported by multiple layers of evidence. Third, downstream transcriptomic, proteomic, CMap, and brain imaging analyses converged on molecular and neurobiological pathways relevant to immune regulation, synaptic function, metabolic processes, and hippocampal structure.

A key observation of this study is the shared genetic background between SC and several drug use traits. Previous studies have reported genetic links between SC and specific medication‐related or disease‐related domains, including antihypertensive drug target genes, cannabis use, and diabetes or insulin resistance (Johnson et al. 2021; Fan and Zhao 2024; D. Y. Lee et al. 2024). Our findings extend these observations by showing that genetic correlations are not restricted to a single drug category but involve multiple medication‐use traits, including drugs used in diabetes, beta blocking agents, agents acting on the renin‐angiotensin system, and HMG CoA reductase inhibitors across both European and East Asian analyses. These correlations should not be interpreted as direct evidence that the medications themselves causally reduce or increase SC risk. Instead, drug use traits in biobank‐scale GWAS often reflect the genetic liability to the underlying medical conditions for which these drugs are prescribed, as well as treatment‐seeking patterns and clinical comorbidity.

Our multi‐trait meta‐analysis identified additional SC‐associated loci beyond previously reported signals. This result is consistent with the rationale of MTAG and related multi‐trait methods, which can increase statistical power by leveraging correlated traits (Turley et al. 2018; Grotzinger et al. 2019; Ray and Chatterjee 2020; Mallard et al. 2022). The identification of novel loci should be interpreted as an expansion of the current SC genetic map rather than a replacement of single‐trait GWAS results. Several loci and genes highlighted in our study overlap with biological themes emphasized by previous SC GWAS, including synaptic biology, neurodevelopment, and immune‐related mechanisms. For example, FURIN has been implicated in SC and neuropsychiatric biology in previous genetic and functional studies (Fromer et al. 2016; Schrode et al. 2019; T. Zhang et al. 2022; Foka et al. 2022). Our findings further support its relevance in a multi‐ancestry and multi‐trait context. At the same time, newly prioritized loci may reflect biological pathways that are more readily detectable when SC is analyzed together with genetically correlated medication‐use phenotypes.

Gene prioritization across FUMA, MAGMA, POPS, TWAS, and SMR provided convergent evidence for a subset of credible genes. This integrative strategy is important because GWAS loci often contain multiple candidate genes, and statistical association alone does not identify the causal transcript or mechanism. The prioritized genes were enriched for synaptic and neurobiological annotations, consistent with the established role of synaptic dysfunction in SC (Wu et al. 2022; Breitmeyer et al. 2023). In addition, several genes pointed to metabolic, immune, and developmental processes. For instance, PCCB has been experimentally linked to SC‐related GABAergic pathways in human forebrain organoid models (W. Zhang et al. 2023), supporting the biological plausibility of our TWAS‐based signal. ARL17B showed cell‐type‐related evidence across brain cell populations, consistent with recent single‐cell studies suggesting that SC risk genes may act through specific neural and glial contexts (Chen et al. 2024; Emani et al. 2024). These findings indicate that SC risk is unlikely to be mediated by a single biological pathway, but rather by coordinated effects across neuronal, immune, metabolic, and developmental systems.

The transcriptomic validation and machine learning analysis further supported the relevance of the prioritized genes, but these results should be interpreted as supportive rather than diagnostic evidence. Among the credible genes, IRF3 showed strong differential expression and contributed to the classification models. IRF3 is a central transcription factor in antiviral and innate immune signaling, which aligns with increasing evidence that immune dysregulation and inflammatory responses may contribute to SC pathophysiology (Dang et al. 2023).

The CMap‐based drug repurposing analysis should be interpreted as a hypothesis‐generating framework rather than direct therapeutic evidence. The identification of cannabinoid receptor modulators is consistent with prior work implicating the endocannabinoid system and cannabis‐related biology in SC risk and symptom regulation (Ballmaier et al. 2007; Obregon et al. 2012; Blaze and Akbarian 2022; Zhuo et al. 2022; Xu and Yang 2022). However, the relationship between cannabinoid signaling and SC is complex, with evidence suggesting both potential therapeutic and risk‐related effects depending on receptor subtype, compound, dose, developmental timing, and clinical context.

The MR analysis linking SC liability with hippocampal subfield volumes is consistent with neuroimaging studies showing structural abnormalities in SC, particularly in hippocampal regions involved in memory, cognition, and psychosis‐related circuitry (Yasuda et al. 2022; Smeland et al. 2018; Lang et al. 2022). The bidirectional associations observed for specific hippocampal subregions suggest that genetic liability to SC and hippocampal morphology may influence each other or share upstream biological determinants.

Several limitations should be considered. First, this study relied on summary‐level GWAS data, which limited the ability to model individual‐level interactions among genetic risk, medication exposure, clinical subtype, and environmental factors. Second, drug use GWAS traits may represent a mixture of medication exposure, underlying disease liability, healthcare access, and prescription behavior, which complicates biological interpretation. Third, although multi‐trait and cross‐ancestry analyses can improve power, they may also introduce heterogeneity due to differences in phenotype definition, LD structure, and sample composition. Fourth, gene prioritization, TWAS, SMR, proteomic association, and CMap analyses are informative but do not establish definitive causality. Experimental validation will be required to determine whether the prioritized genes and compounds directly influence SC‐relevant cellular phenotypes.

In sum, our findings elucidate the complex genetic architecture of SC, providing a basis for further functional studies and clinical applications. Integrating broader data dimensions and functional analyses on genetic variations' impact on disease phenotypes and treatment responses will pave the way for uncovering new mechanisms and therapeutic targets.

## Author Contributions

**Haili Wang**: data curation, writing – original draft, validation. **Ping Yan**: conceptualization, writing – original draft, data curation, formal analysis. **Rui Xie**: validation, visualization. **Songnian Fu**: conceptualization, writing – review and editing, validation, supervision. **Ye Shu**: visualization, formal analysis. **Ainiwaner Reyidan**: writing – original draft, validation.

## Conflicts of Interest

The authors declare no conflicts of interest.

## Supporting information




**Supplementary Figure**: brb371594‐sup‐0001‐FigureS1.pdf

**Supplementary Table**: brb371594‐sup‐0002‐TableS1.xlsx

## Data Availability

LD data were derived from the 1000 Genomes Project (https://www.internationalgenome.org/). MAGMA gene IDs and locations were extracted from https://ctg.cncr.nl/software/magma. eQTL data were from YangLab (https://yanglab.westlake.edu.cn/software/smr/#eQTLsummarydata). The gene feature repository used for POPS was from https://github.com/FinucaneLab/gene_features. LDSC: https://github.com/bulik/ldsc. MTAG: https://github.com/JonJala/mtag. METAL: https://github.com/statgen/METAL. MAGMA: https://ctg.cncr.nl/software/magma. POPS: https://github.com/FinucaneLab/pops. SMR: https://yanglab.westlake.edu.cn/software/smr/#Overview. BLISS: https://github.com/gcb‐hub/BLISS. Popcorn: https://github.com/brielin/Popcorn. TWAS: http://gusevlab.org/projects/fusion/.
