# Supplementary material for: Multi‐Trait Genetic Insights Into Schizophrenia Across Ancestries: Genome‐Wide Association Meta‐Analyses, Machine Learning, and Drug Repurposing Study
Source: Brain Behav. 2026 Jul 9;16(7):e71594. doi: 10.1002/brb3.71594 (PMC13347149; doi:10.1002/brb3.71594)
Supplement: Supplementary file 1 — Supplementary Figure: brb371594‐sup‐0001‐FigureS1.pdf [file BRB3-16-e71594-s002.pdf]

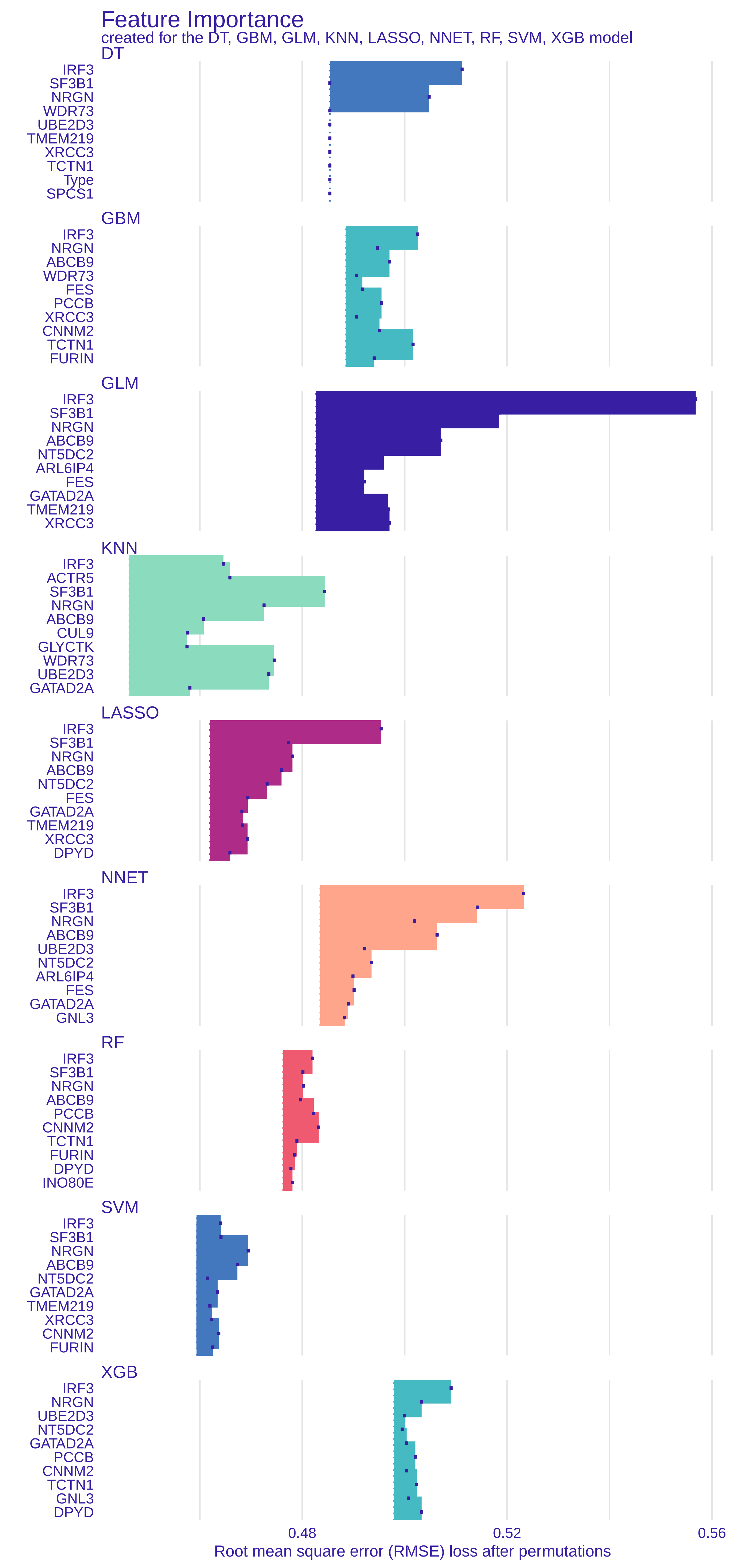
Feature Importance
created for the DT, GBM, GLM, KNN, LASSO, NNET, RF, SVM, XGB model
DT
IRF3
SF3B1
NRGN
WDR73
UBE2D3
TMEM219
XRCC3
TCTN1
Type
SPCS1
GBM
IRF3
NRGN
ABCB9
WDR73
FES
PCCB
XRCC3
CNNM2
TCTN1
FURIN
GLM
IRF3
SF3B1
NRGN
ABCB9
NT5DC2
ARL6IP4
FES
GATAD2A
TMEM219
XRCC3
KNN
IRF3
ACTR5
SF3B1
NRGN
ABCB9
CUL9
GLYCTK
WDR73
UBE2D3
GATAD2A
LASSO
IRF3
SF3B1
NRGN
ABCB9
NT5DC2
FES
GATAD2A
TMEM219
XRCC3
DPYD
NNET
IRF3
SF3B1
NRGN
ABCB9
UBE2D3
NT5DC2
ARL6IP4
FES
GATAD2A
GNL3
RF
IRF3
SF3B1
NRGN
ABCB9
PCCB
CNNM2
TCTN1
FURIN
DPYD
INO80E
SVM
IRF3
SF3B1
NRGN
ABCB9
NT5DC2
GATAD2A
TMEM219
XRCC3
CNNM2
FURIN
XGB
IRF3
NRGN
UBE2D3
NT5DC2
GATAD2A
PCCB
CNNM2
TCTN1
GNL3
DPYD
0.48
0.52
0.56
Root mean square error (RMSE) loss after permutations
